# Veterinary Medical Society of the University of Pennsylvania

**Published:** 1898-02

**Authors:** M. Jacob

**Affiliations:** Secretary


Secretary.

### VETERINARY MEDICAL SOCIETY OF THE UNIVERSITY OF PENNSYLVANIA.

THE meetings held during the month of January were of special interest, as the subjects for debate and the papers to be read were well selected, and all the members seemed to be working with a common effort to bring every part up to the standard. The money taken in by the Treasurer during the last meeting amounted to eight dollars and fifty cents. Mr. Stehle's name was proposed and duly elected a member of the Society.

One of the most important changes in the Society was the election of new officers, which resulted as follows: Honorary President, Prof. J. W. Adams. He was unanimously elected to succeed Prof. R. S. Huidekoper. President, J. E. Spindler; Vice-President, G. Chesley; Treasurer, S. Blount; Librarian, E. Newcomer; Executive Committee, S. McClure, '98; H. Hoopes, '99; J. P. Miller, '99; E. Cornman, 1900.

Some of the interesting and instructive papers read to the Society were: "Etiology of Epizoötic Abortion," by Prof. S. J. J. Harger. He traced the full course of the disease, and the members present were greatly benefited by the paper.

Mr. E. Newcomer presented the Society with an excellent paper; he traced the horse from its first knowledge in history until the present time; he showed some of the important changes that have taken place in the equine family, the most important of which was the great development of speed. The paper was highly appreciated by the members present.

Mr. Hoopes read a very interesting article on "What Parts have the Arabian, Turkish and Barbary Horses Played in the Foundation of the Racing Breed of Horses." Mr. Hoopes thought that subjects of this class were very important for the veterinary student to be well versed upon, as in after life he will constantly come across instances in which he will be called upon to discuss this question at some institute or Grangers' meeting, where such a subject, if well delivered, would receive the highest appreciation.

The subjects for debate during this month's meetings were as follows:

*Resolved*, That veterinarians should be active members of humane societies. Affirmative—Mr. Megary, Mr. Taylor, Mr. White. Negative—Mr. Murphy, Mr. Miller, Mr. Beaver.

The judges, Messrs. Kirby, Newcomer, and Hughes, decided in favor of the negative.

*Resolved*, That the United States Government should give the veterinarian in the United States Army the rank and pay of second lieutenant.

Affirmative—Mr. Spaethe, Mr. Jacobs, Mr. Nolan. Negative—Mr. Murphy, Mr. Townsend, Mr. Reardon.

Mr. Spindler made a report in behalf of the supper committee, it having liquidated the expenses and obtained a receipt in full. It was moved and seconded to offer a vote of thanks to the committee, which was very heartily adopted.

It was also moved and seconded to offer a vote of thanks to the outgoing officers; carried.

The Society adjourned at 10.15 P.M.

M. JACOB,
Secretary.

